# Transmissible endoplasmic reticulum stress from myocardiocytes to macrophages is pivotal for the pathogenesis of CVB3-induced viral myocarditis

**DOI:** 10.1038/srep42162

**Published:** 2017-02-08

**Authors:** Hui Zhang, Yan Yue, Tianle Sun, Xuejie Wu, Sidong Xiong

**Affiliations:** 1 Jiangsu Key Laboratory of Infection and Immunity, Institutes of Biology and Medical Sciences, Soochow University, Suzhou, P.R. China

## Abstract

Infiltrating macrophages have been proven as a pivotal pathological inflammatory cell subset in coxsackievirus B3 (CVB3) induced viral myocarditis. However, the mechanisms underlying the initiation and promotion of macrophage pro-inflammatory responses are still blur. We previously reported that cardiac ER stress contributed to CVB3-induced myocarditis by augmenting inflammation. In this study, we focused on the influence of ER stress on the macrophage inflammatory responses in the viral myocarditis. We found that ER stress was robustly induced in the cardiac infiltrating macrophages from CVB3-infected mice, and robustly facilitated the production of pro-inflammatory cytokines (IL-6, IL-12, MCP-1 and IP-10). Consistently, adoptive transfer of ER stressed macrophages significantly worsened the viral myocarditis; while transfer of ER stress-inhibited macrophages obviously alleviated the myocarditis. To our surprise, this significantly activated ER stress was not directly caused by the virus stimulation, but was transferred from the CVB3-infected, ER stressed myocardiocytes via soluble molecules in a TLR2, 4-independent way. In the present study, we reported that the transmissible ER stress from the infected myocardiocytes to macrophages could augment the pro-inflammatory responses and promoted the pathogenesis of viral myocarditis. Blocking ER stress transmission, instead of inhibiting its initiation, may represent novel therapeutic strategies against viral myocarditis.

Myocarditis, characterized by an inflammatory cellular infiltrate in heart tissues, is one main cause of sudden death in adolescence and adults less than 40^1^,^2^,^3^. Enteroviruses including coxsackievirus B3 (CVB3) are identified as the main etiological agents of viral myocarditis^3^,^4^, and associated with up to 45% of human myocarditis and dilated cardiomyopathy^2^,^5^. With the aid of murine CVB3-induced myocarditis models which share many characteristics with human viral myocarditis, it has been demonstrated that immune-mediated indirect myocardial injury, instead of virus-mediated direct destroy, is the primary cause of myocardial inflammation and necrosis^3^,^6^. Accordingly, various strategies based on immune manipulation, modulation, or suppression have been developed and showed promising protection effects against severe viral myocarditis in mice^7^,^8^,^9^. Therefore, elucidating the initiation and augmentation mechanisms underlying the excessive immune responses would not only help understanding the disease pathogenesis, but also might facilitate the development of new therapeutic recipes against CVB3-induced myocarditis.

As an essential innate immune cell component, cardiac macrophages begin to enrich at as early as day 3 post CVB3 infection, and represent the dominant infiltrating inflammatory cells^10^. Conditioned by the infected cardiac microenvironment, infiltrating macrophages appear the classically activated phenotypes (M1-type) and promote cardiac inflammation by secreting pro-inflammatory cytokines such as TNF-α, IL-6^11^,^12^,^13^. More importantly, these M1-type macrophages could further dictate the subsequent adaptive immunity to the pathological Th1 responses and collaboratively aggravate myocarditis^14^. In support of this, depletion of macrophages significantly improve both acute and chronic viral myocarditis, further confirming the critical pathological role of macrophages in CVB3-induced myocarditis^11^,^15^. However, so far the mechanisms endowing the cardiac infiltrating macrophages with pro-inflammatory properties are still not fully understood.

Endoplasmic reticulum (ER) is an essential organelle responsible for protein synthesis, folding, and modification. Stimuli perturbing ER homeostatic would cause stress and activate the subsequent unfolded protein response (UPR) consisting of three highly coordinated signaling cascades: IRE1α (inositol-requiring 1α), PERK (PKR-like ER kinase) and ATF6 (activating transcription factor 6)^16^,^17^. Upon activation, these three ER stress sensors would disassociate from 78 kD aglucose-regulated protein (GRP78) and modulate the down-stream transcriptional and translational machineries. Recently, a line of evidence revealed the crosstalk between ER stress and tissue inflammation. Kaser A and colleagues^18^ reported that depletion of X-box binding protein 1 (XBP1), a key transcription factor in IRE1-associated pathway, in epithelial cells led to the spontaneous enteritis and increased susceptibility to colitis. In addition to impacting tissue cells, ER stress also directly manipulates immune cell activities. For example, in combination with toll-like receptor (TLR) agonists, ER stress can significantly increase the production of IL-23 by dendritic cells^19^, remarkably enhance the production of inflammatory cytokine (IL-6, IL-23) by macrophages^20^ and drive the processing of pro-IL-1β in response to LPS stimulation^21^. In addition, ER stress is also responsible for the differentiation of alternatively activated macrophages in the context of atherosclerosis^22^. Considering that cardiac ER stress substantially contributes to viral myocarditis by augmenting inflammation and promoting myocardiocytes apoptosis^23^,^24^, and infiltrating macrophages are critical pathological inflammatory cells in viral myocarditis^11^,^15^,^25^, herein we hypothesized that ER stress might occur in the cardiac infiltrating macrophages in the context of CVB3 myocarditis and impact the pro-inflammatory responses of macrophages.

In this study, we showed that ER stress was robustly activated in the cardiac infiltrating macrophages from CVB3-infected mice, and obviously promoted the production of pro-inflammatory cytokines (IL-6, IL-12, MCP-1 and IP-10). Adoptive transfer of ER stressed macrophages to CVB3-infected mice significantly aggravated the viral myocarditis. Interestingly, we found that this potent macrophages ER stress was not directly caused by CVB3 stimulation, but was transferred from the CVB3-infected, ER-stressed myocardiocytes via soluble molecules. Take together, our study suggested that the transmissible ER stress from CVB3-infected myocardiocytes endowed the cardiac infiltrating macrophages with pro-inflammatory properties, and contributed greatly to the pathogenesis of viral myocarditis.

## Results

### Cardiac ER stress significantly aggravated CVB3-induced viral myocarditis

**Figure 1:**
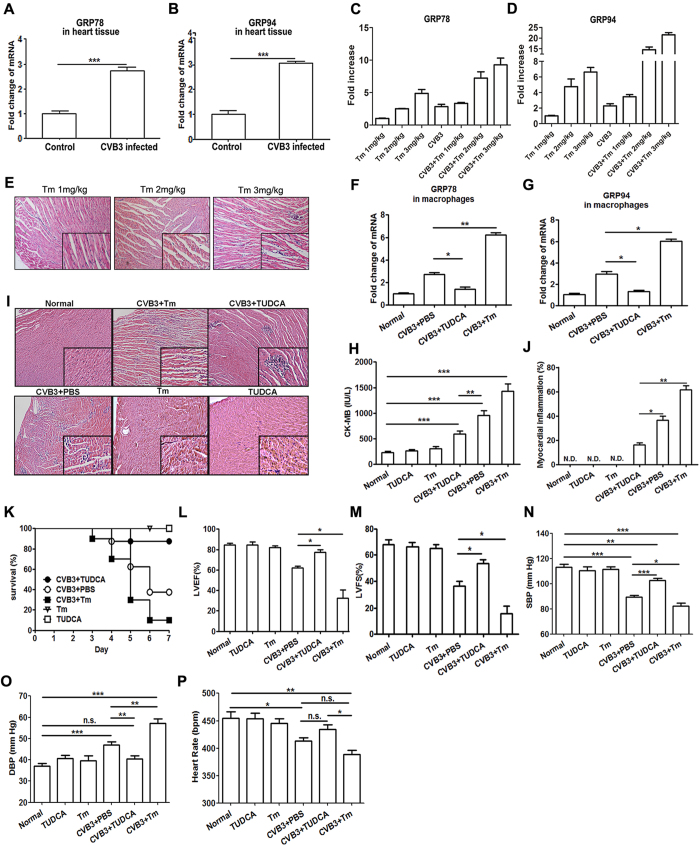
ER stress aggravated CVB3-induced myocarditis. At day 7 post infection, cardiac expression of (**A**) GRP78 and (**B**) GRP94 was determined by real-time PCR assays. Optimized the dosage of Tm (**C–E**). Mice were treated with 2 mg/kg Tm or 200 mg/kg TUDCA, and cardiac expression of (**F**) GRP78 and (**G**) GRP94 was determined by real-time PCR. Meanwhile, the severity of viral myocarditis was evaluated by the following indices: (**H**) serum CK-MB activity (**I**) pathological observation of heart tissue (**J**) percentage of myocardial inflammation (**K**) survival rates in a 7-day period (**L**) left heart ejection fraction (LVEF) (**M**) left heart fractional shortening (LVFS) (**N**) systolic blood pressure (SBP) (**O** diastolic blood pressure (DBP) and (**P**) heart rate (HR). Each group contained 5 mice. For survival rate evaluation, each group contained 10 mice. Individual experiment was conducted 3 times with similar results. *P < 0.05, **P < 0.01, ***P < 0.001.

Mice were infected with 10^3^ plaque-forming unit (PFU) CVB3 at day 0 and the induction of cardiac ER stress was monitored at day 7 by detecting the expression of its specific markers GRP78 and GRP94. As shown in Fig. 1A,B, obviously increased GRP78 and GRP94 were seen in the hearts of CVB3-infected mice compared with the control group, indicating the robust induction of cardiac ER stress. To further elucidate the role of ER stress in the development of viral myocarditis, CVB3-infected mice were treated with 100 mg/kg ER stress inhibitor tauroursodeoxycholic acid (TUDCA) or 2 mg/kg activator tunicamycin (Tm) at day 1 and day 4 post infection, and the expression of GRP78 and GRP94 in mice heart tissue were detected by real-time PCR (Fig. 1F,G), and then the changes in myocarditis severity were determined. It was found that compared with the control infected group, mice treated with TUDCA showed significantly reduced CK-MB activity (590 vs 967, Fig. 1H), relieved myocardial inflammation (Fig. 1I,J), an increased the survival rate (90% vs 40%, Fig. 1K) and obviously improved left heart ejection fraction (LVEF) and left heart fractional shortening (LVFS) (Fig. 1L,M). And mice systemic hemodynamic parameters were evaluated by the indices of systolic blood pressure (SBP), diastolic blood pressure (DBP) and heart rate (HR). As shown in Fig. 1N,O,P, mice treated with TUDCA showed significantly higher SBP (102.7 vs 89.3, P < 0.001) and lower DBP (40.5 vs 47, P < 0.01) than infected control mice. All these data indicated the remission of viral myocarditis. In contrast, mice treated with Tm exhibited substantially aggravated myocarditis (Fig. 1I–P).

### ER stressed cardiac macrophages played a pivotal pathological role in CVB3-induced myocarditis

**Figure 2:**
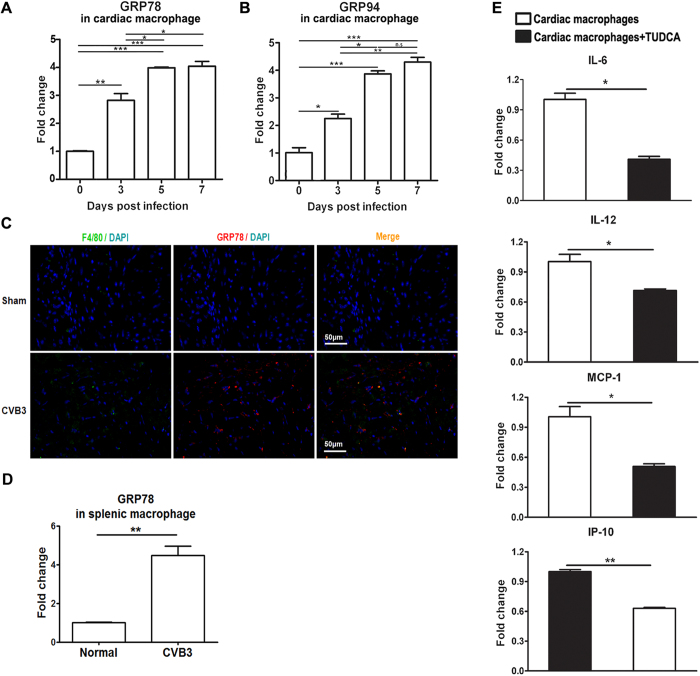
ER stress occurred in the cardiac infiltrating macrophages and facilitated the pro-inflammatory cytokine production. (**A**) Dynamic expression of GRP78 in the cardiac macrophages post infection. (**B**) Dynamic expression of GRP94 in the cardiac macrophages post infection. (**C**) Immunofluorescence analysis of the induction of ER stress in macrophages derived from CVB3-infected mice. Heart sections from sham-infected mice (upper panels) or CVB3-infected mice (lower panels) were stained with Cy3-labeled rabbit anti-mouse GRP78 (red) or FITC-labeled rat anti-mouse F4/80 (green), and DAPI identifying the nucleus (blue). Images are representative of at least 3 independent determinations. (**D**) Splenic macrophages were isolated at day 7 post infection and the expression of GRP78 was detected by real-time PCR. (**E**) Cardiac macrophages were isolated at day 7 post infection and treated with TUDCA, the production of pro-inflammatory cytokines (IL-6, IL-12, MCP-1 and IP-10) was determined by real-time PCR. Each group contained 5–6 mice. Individual experiment was conducted 3 times with similar results. *P < 0.05, **P < 0.01, ***P < 0.001.

**Figure 3:**
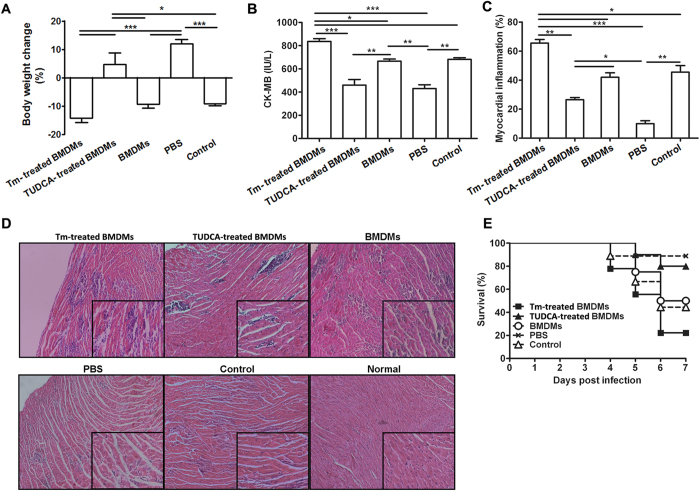
ER stressed macrophages obviously aggravated CVB3-induced myocarditis. After depleting macrophages, mice were infected CVB3 at day 0 and received Tm-treated BMDMs, TUDCA-treated BMDMs, BMDMs or PBS at day 3, and the severity of viral myocarditis was monitored by body weight loss (**A**), serum CK-MB activity (**B**), percentage of myocardial inflammation (**C**), pathological observation of heart tissues (**D**) and survival rates (**E**). CVB3-infected mice without macrophage depletion were applied as a control group. Each group contained 4–5 mice. For survival rate evaluation, each group contained 10 mice. Individual experiment was conducted 3 times with similar results. *P < 0.05, **P < 0.01, ***P < 0.001.

As the earliest infiltrating and predominant inflammatory cells, cardiac macrophages have been proven to contribute greatly to the development of viral myocarditis, however, it is still unclear whether the cardiac ER stress could influence the inflammatory responses of infiltrating macrophages. Here, we detected the dynamic activation of ER stress in the cardiac infiltrating macrophages from CVB3 infected mice, and found that macrophages began to undergo obvious ER stress even at the very beginning of enrichment in heart tissues (day 3 post infection, Fig. 2A,B)^11^. As time passed by, the extent of macrophage ER stress was further increased, and reached the peak at day 5 and remained the plateaus level at day 7. Of note, these dynamic changes of macrophage ER stress were consistent with the progress and magnitude of viral myocarditis^11^. The results were further confirmed by the study of *in situ* immunofluorescence, which showed that cardiac GRP78 expression was obviously induced after CVB3 infection, confirmed that ER stress was efficiently induced in the CVB3-infected hearts. More importantly, F4/80^+^ cells co-localized with the expression of ER stress marker GRP78, indicating that infiltrating macrophages underwent ER stress (Fig. 2C). Interestingly, inhibition of the ER stress in the cardiac macrophages could potentially decrease the production of pro-inflammatory cytokines (IL-6, IL-12, MCP-1 and IP-10) (Fig. 2D), indicating that ER stress amplified the pro-inflammatory responses of cardiac macrophages. To further validate the role of ER stressed macrophages in viral myocarditis, ER-stressed or ER stress-inhibited bone marrow-derived macrophages (BMDMs) were prepared respectively, and adoptively transferred into the macrophage-depleted recipients at day 3 post CVB3 infection. It was found that compared with the control mice receiving BMDMs, mice receiving ER stressed BMDMs succumbed to more severe viral myocarditis, as reflected by the more body weight loss, increased serum CK-MB, exacerbated pathological changes and a lower survival rate (Fig. 3). Conversely, mice receiving ER stress-inhibited BMDMs exhibited obviously alleviated myocarditis (Fig. 3). All these data revealed that ER stressed macrophages exerted potent pathological effects on CVB3-induced viral myocarditis.

### Macrophage ER stress was not directly induced by CVB3 stimulation, but was transferred from CVB3-infected, ER stressed myocardiocytes

**Figure 4:**
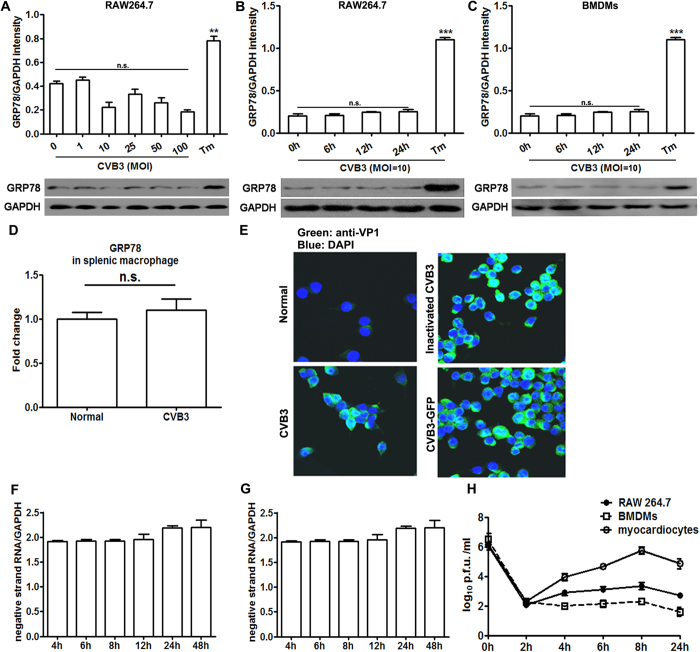
CVB3 stimulation could not directly elicit ER stress in macrophages. (**A**) GRP78 expression in RAW264.7 cells after stimulated with various doses of CVB3 for 24 h. (**B**) GRP78 expression in RAW264.7 cells after stimulated with CVB3 at a dose of 10 MOI for various hours. (**C**) GRP78 expression in BMDMs after stimulated with CVB3 at a dose of 10 MOI for various hours. (**D**) GRP78 expression in splenic macrophages after stimulated with CVB3 at a dose of 10 MOI *in vitro* for 24 h. RAW264.7 cells were infected with CVB3, inactivated CVB3 or a recombinant infectious CVB3 expressing GFP (CVB3-GFP) respectively at a dose of MOI = 10 for 24 h, and following removal of the inoculum, cells were collected and the level of CVB3 structural protein VP1 was detected by immunofluorescence assays (**E**), and the amounts of positive- (**F**) and negative-(**G**) strand RNAs of CVB3 were detected by real-time PCR. RAW264.7 cells, BMDMs or myocardiocytes were infected with CVB3 at 10 p.f.u. per cell and following removal of the inoculum, amounts of progeny virus were determined over 24 h (**H**). Individual experiment was conducted 3 times with similar results. **P < 0.01, ***P < 0.001, n.s., no significance.

Since ER stressed macrophages took part in the pathogenesis of viral myocarditis, it is important to decipher the initiation mechanism of macrophage ER stress. It was found that *in vitro* stimulation of murine macrophage cell line RAW264.7 cells with CVB3 at various doses (ranging from 1 to 100 multiplicity of infection (MOI)) could not induce obvious ER stress responses (Fig. 4A). Even extending the CVB3-stimulation time scale to 24 h, no obviously up-regulated GRP78 was observed either in RAW264.7 cells (Fig. 4B) or in BMDMs (Fig. 4C). In contrast, these macrophages underwent robust ER stress in response to Tm treatment. We also stimulated splenic macrophages of naive mice with CVB3 *in vitro*, no detectable UPR activation was detected (Fig. 4D), suggesting that CVB3 couldn’t infect macrophages. All these data suggested that macrophage ER stress was not directly initiated by virus stimulation, but by other factors.

**Figure 5:**
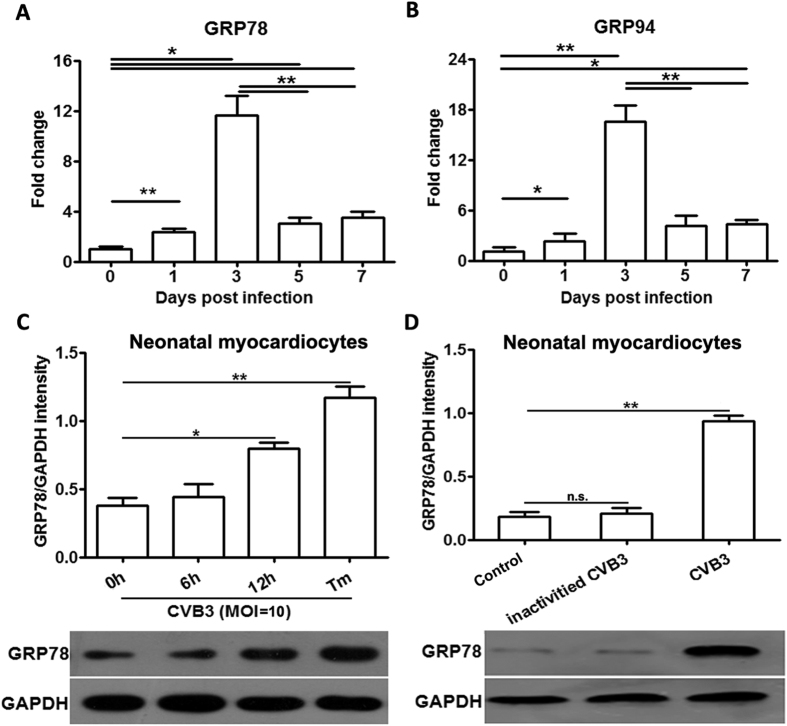
CVB3 infection efficiently induced ER stress in myocardiocytes. Mice were infected with CVB3 at day 0, and myocardiocytes were isolated at indicated time point, and the dynamic expression of GRP78 (**A**) and GRP94 (**B**) in infected myocardiocytes was analyzed with real-time PCR assays. Each group contained 4–5 mice. In addition, neonatal myocardiocytes were prepared and infected with CVB3 for different hours (**C**), or infected with CVB3 or inactivated CVB3 for 12 h (**D**), the GRP78 expression was monitored by real-time PCR assays. Individual experiment was conducted 3 times with similar results. *P < 0.05, **P < 0. 01.

As previous literature showed that in the context of tumor microenvironment, ER stress could be transferred from tumor cells to macrophages, and then endowed the latter with a pro-inflammatory phenotype^20^. Inspired by this study, we assumed that ER stress in the cardiac macrophages might be also transferred from the surrounding tissue cells (myocardiocytes) in the condition of CVB3 myocarditis. To testify this hypothesis, we dynamically detected the myocardiocyte ER stress in the infected mice, and found that the expression of GRP78 and GRP94 appeared to increase (about 2-fold) at day 1 post infection and peaked at day 3 (about 10- and 16-fold, respectively), but then robustly decreased at day 5 and day 7 (Fig. 5A,B), suggesting that the infected myocardiocytes underwent ER stress. In line with these *in vivo* data, *in vitro* infection of freshly isolated neonatal myocardiocytes also substantially induced ER stress (Fig. 5C), and this effect seemed dependent on the infectivity of CVB3 virus, as it was completely deprived once CVB3 was inactivated (Fig. 5D).

**Figure 6:**
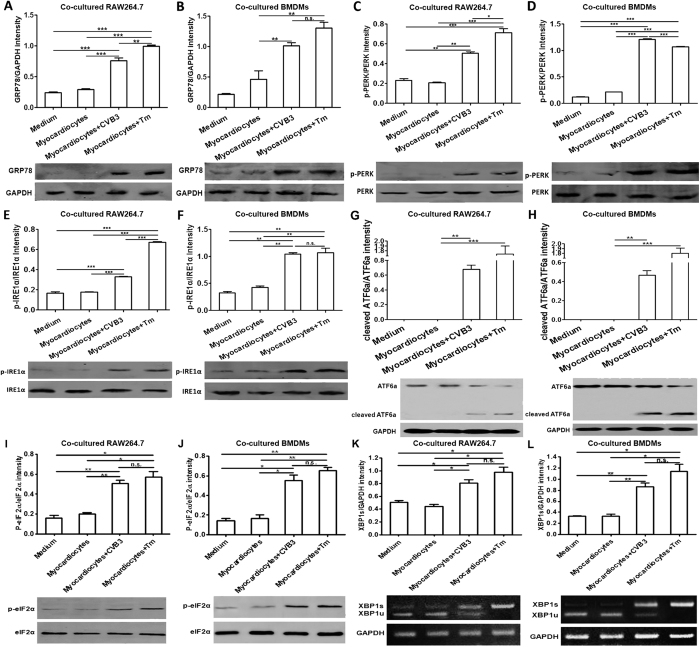
Macrophages underwent ER stress upon co-cultured with CVB3-infected, ER stressed myocardiocytes. RAW264.7 cells or BMDMs were co-cultured with myocardiocytes and then infected with CVB3 (10 MOI) for 24 h, then GRP78 expression in co-cultured RAW264.7 (**A**) or BMDMs (**B**) was determined by western blot. Meanwhile, the expression of phosphorylated PERK, IRE1α and eIF2α in RAW264.7 cells (**C**,**E**,**I**) or BMDMs (**D**,**F**,**J**), and the cleavage of ATF6 in RAW264.7 cells (**G**) or BMDMs (**H**) were also detected by western blot assays. XBP1 mRNA splicing in RAW264.7 cell lines (**K**) or BMDMs (**L**) were detected by RT-PCR. Individual experiment was conducted 3 times with similar results. *P < 0.05, **P < 0.01, ***P < 0.001.

To determine whether ER stress could be transferred from the infected myocardiocytes to the infiltrating macrophages, macrophages were co-cultured with the infected myocardiocytes for 24 h, and then the induction of ER stress was determined. As shown in Fig. 6, compared with the control macrophages, macrophages co-cultured with the CVB3-infected myocardiocytes or Tm-treated myocardiocytes expressed much higher levels of GRP78, phospharylated IRE1α, eIF2α and PERK, and obviously ATF6 cleavage. In addition, we also detected expression of the spliced form of Xbox-binding protein 1(XBP1s) mRNA by RT-PCR, the data showed that the spliced XBP1 significantly upregulated in the macrophages (RAW264.7 cell lines and BMDMs) co-cultured with CVB3-infected myocardiocytes in comparison to the counterparts co-cultured with non-infected myocardiocytes (Fig. 6K,L). All these data indicated the efficient induction of all of the three UPR arms. These data revealed that macrophage ER stress could be transferred from CVB3-infected, ER stressed myocardiocytes.

### ER stress transmission from the infected myocardiocytes to macrophages was mainly mediated by soluble molecules, but not cell-cell contact way

**Figure 7:**
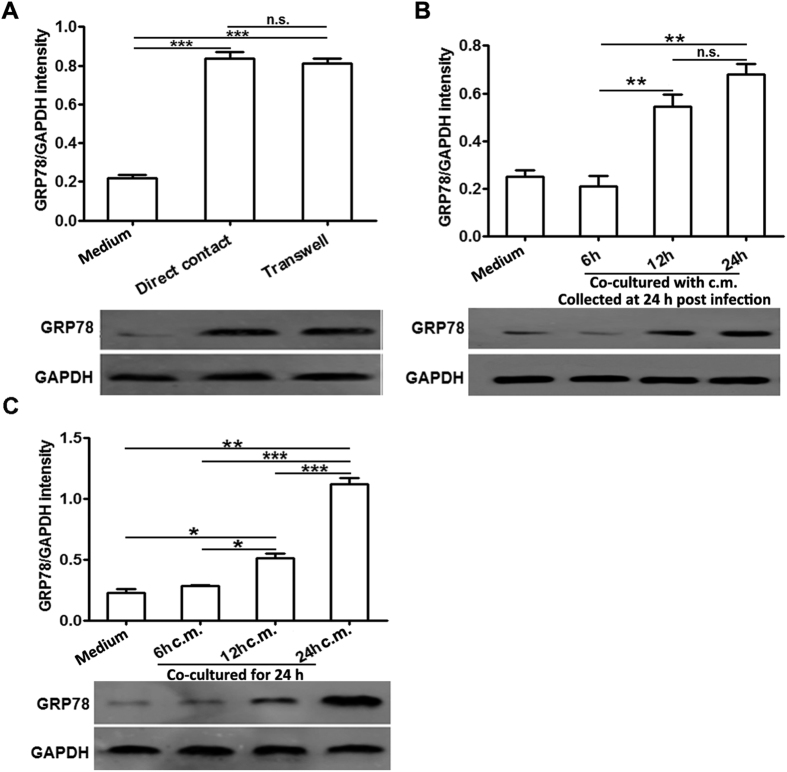
ER stress transmission to receiver macrophages was mainly mediated by the conditioned medium of infected myocardiocytes. (**A**) RAW264.7 and myocardiocytes were plated together or separated by trans-well membrane, infected with CVB3 (10 MOI) for 24 h, and then GRP78 expression in RAW264.7 cells was detected by western blot assays. (**B**) RAW264.7 cells were cultured for 6, 12 or 24 h with myocardiocyte-derived conditioned medium (c.m.) which was collected at 24 h post infection, and then GRP78 expression in RAW264.7 cells was detected by western blot assays. (**C**) RAW264.7 cells were cultured for 24 h with myocardiocyte-derived conditioned medium (c.m.) which was collected at various time points (6, 12 or 24 h) post infection, and then GRP78 expression in RAW264.7 cells was detected by western blot assays. Individual experiment was conducted 3 times with similar results. *P < 0.05, **P < 0.01, ***P < 0.001, n.s., no significance.

To determine whether cell-cell contact was required for this ER stress transmission, infected myocardiocytes and macrophages were co-cultured on the opposite sides of a porous transwell membrane insert. As illustrated in Fig. 7A, disrupting cell-cell contact did not obviously impede ER stress transmission between the co-cultured infected myocardiocytes and macrophages, suggesting that soluble molecules in the conditioned medium were mainly responsible for this ER stress transmission. These results were further confirmed by the efficient induction of ER stress in the macrophages co-cultured with the infected myocardiocyte-derived conditioned medium. It was founded that GRP78 protein appeared to be up-regulated at as early as 12 h post co-culture and further increased at 24 h (Fig. 7B). We further investigated the ER stress-transferring abilities of various conditioned medium derived from the infected myocardiocytes, which was collected at different time-points (6, 12 and 24 h) post infection, and found that only the conditioned medium collected at 12 and 24 h possessed this ER stress-transferring ability, while the medium collected at 6 h did not. These results were consistent with the dynamics of ER stress induction in the CVB3-infected myocardiocytes, in which GRP78 was obviously up-regulated at 12 h, but not 6 h post infection (Fig. 5C). Taken together, these data indicated that the conditioned medium, but not cell-cell contact, was operative in the ER stress transmission between the infected myocardiocytes and macrophages.

### Transferred ER stress robustly amplified the production of pro-inflammatory cytokines

**Figure 8:**
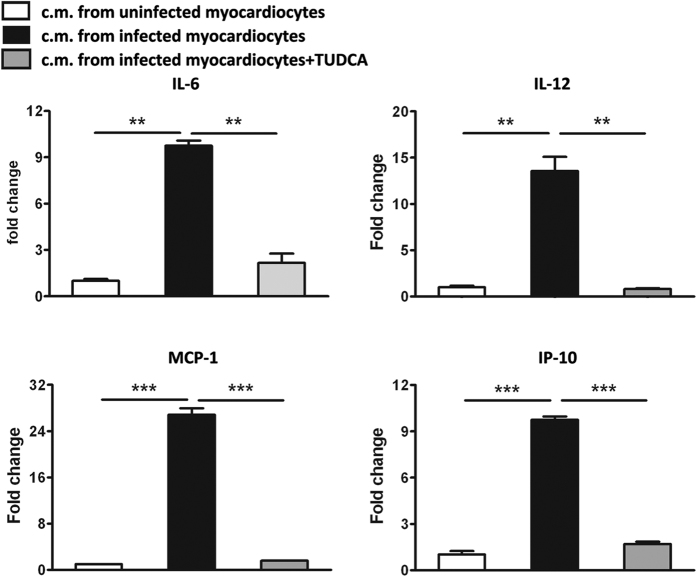
Transmissible ER stress endowed macrophages with the pro-inflammatory phenotype. RAW264.7 cells were cultured for 24 h with conditioned medium (c.m.) from infected myocardiocyte, followed by treating with or without TUDCA for 24 h, and then production of pro-inflammatory cytokines (IL-6, IL-12, MCP-1 and IP-10) was detected by real-time PCR assays. Individual experiment was conducted 3 times with similar results. **P < 0.01, ***P < 0.001.

We next detected whether this transmissible ER stress could impact the pro-inflammatory property of macrophages, and found that compared with the control cells co-cultured with the uninfected myocardiocyte-derived conditioned medium, macrophages co-cultured with the infected myocardiocyte-derived conditioned medium produced much higher levels of IL-6, IL-12, MCP-1 and IP-10. Consistently, treating co-cultured macrophages with TUDCA could robustly decrease the production of these pro-inflammatory cytokines (Fig. 8), further confirming the inflammation-promoting effect of the transmissible ER stress. These results were also consistent with the *in vivo* data as shown in Figs 2C and 3, in which ER stress facilitated the pro-inflammatory phenotypes of cardiac infiltrating macrophages and contributed to the development of CVB3-induced viral myocarditis.

### ER stress was transferred from infected myocardiocytes to macrophages in a TLR2/4-independent way

**Figure 9:**
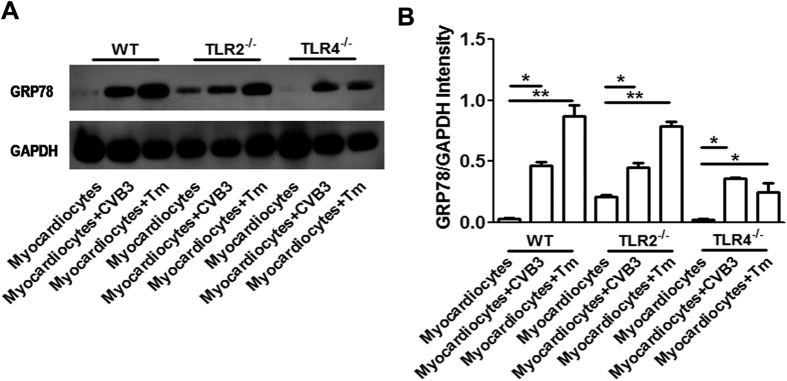
ER stress transmission between infected myocardiocytes and macrophages was independent on TLR2/4 pathways. BMDMs derived from wild type (WT), TLR2^−/−^ or TLR4^−/−^ mice were cultured with conditioned medium (c.m.) from uninfected, CVB3-infected or Tm-treated myocardiocytes for 24 h, and then expression of GRP78 in co-cultured macrophages was detected by western blot assays. (**A**) Expression of GRP78 protein in co-cultured macrophages was detected by western blot assays (**B**) Semi-quantification of co-cultured macrophages GRP78 expression. Individual experiment was conducted 3 times with similar results. *P < 0.05, **P < 0.01.

Considering that TLR4 signaling pathway has been proven to be involved in the ER stress transmission from tumor cells to macrophages^20^, therefore herein we explored the roles of TLR2 and TLR4 in the ER stress transmission between infected myocardiocytes and macrophages. After co-culturing BMDMs derived from TLR2^−/−^ or TLR4^−/−^ mice with uninfected, CVB3-infected or Tm-treated myocardiocytes for 24 h, the GRP78 expression in these co-cultured macrophages was determined by western blot assays. As shown in Fig. 9, GPR78 expression was significantly up-regulated not only in wild type macrophages, but also in TLR2^−/−^ and TLR4^−/−^ macrophages, indicating that this transmission of ER stress was independent on TLR2 or TLR4 pathways.

## Discussion

ER stress generally triggered by accumulated mis-folded proteins was initially shaped as a serial of host compensatory responses to adapt and recover ER homeostasis. If the stress is persistent and strong, ER stress-associated apoptosis signaling pathways will be activated. In the context of CVB3 infection, ER stress-mediated apoptosis has been evidenced^24^. In addition to its effect on cell survival, ER stress also possesses immune modulation capability and participates in many infectious, metabolic and immune pathological diseases^26^,^27^,^28^,^29^. It not only facilitates the activation of innate immunity^30^, promotes pro-inflammatory cytokine production^31^, but also contributes to macrophage polarization as well as foam cell formation^22^. Since ER stress aggravates CVB3-induced viral myocarditis^23^, and macrophages represent one of the most important inflammatory cell subset in CVB3-infected mice, herein we emphatically studied the initiation mechanism of macrophage ER stress as well as its impact on the process of viral myocarditis.

It was found that the cardiac infiltrating macrophages underwent potent ER stress at day 3 post CVB3 infection, and continued to increase as disease progressed. When inhibiting ER stress, the production of pathological pro-inflammatory cytokines by the cardiac infiltrating macrophages was substantially reduced, indicating the blunted pro-inflammatory phenotype of macrophages. The pathological role of ER stressed macrophages in CVB3-induced myocarditis was further confirmed by the adoptive transfer of Tm pre-treated macrophages into the macrophage-depleted recipient mice at day 3 post infection.

Next, we tried to elucidate the trigger of macrophage ER stress in CVB3-induces myocarditis, and surprisingly found that the virus stimulation could not directly induce macrophage ER stress. Then we detected whether CVB3 could infect macrophages, both of our and other groups^32^ observed that although CVB3 virus particles can enter cells (Fig. 5E), but they can not efficiently replicate there (as shown by the limited amount of positive- and negative-strand RNAs of CVB3, Fig. 5F,G), so the amount of released virus particles is very limited (Fig. 5H). This deficiency in supporting virus replication might account for the macrophage resistance for CVB3-induced UPR. In other words, the activation of CVB3-induced UPR might depend on abundant intracellular production of virus replication or abundant amount of viral load. Therefore, the ER stress of macrophages in CVB3-infected mice hearts was more likely to be launched by the cardiac microenvironment factors, but not by virus alone. In support of our hypothesis, Mahadevan NR *et al*.^20^ demonstrated that ER stress could be transferred from tumor cells to macrophages, and promoted the production of pro-inflammatory cytokines. In this study, we did observe that the myocardiocyte ER stress was induced at as early as day 1 post CVB3 infection and peaked at day 3, however, it dramatically decreased at day 5 and day 7. This dynamic was inconsistent with the process of myocarditis in which cardiac inflammation and injury begin to appear at day 3, and peak at day 7, indicating that the myocardiocyte ER stress might not directly contribute to the cardiac inflammation. Of note, the peak time of myocardiocyte ER stress happened to coincide with the beginning time of macrophage enrichment in hearts, indirectly suggesting that myocardiocyte ER stress might be associated with the immune responses of macrophages. In line with these *in vivo* data, *in vitro* results also revealed that CVB3 infection could efficiently induce ER stress in myocardiocytes. Inspired by all of these, we deduced that in the condition of CVB3-induced myocarditis, ER stress in cardiac infiltrating macrophages might be transferred from infected, ER stressed myocardiocytes. As it turned out that co-culture with infected myocardiocytes could obviously induce ER stress in macrophages and significantly promote the production of pro-inflammatory cytokines. This ER stress transmission process was mainly dependent on the conditioned medium of infected myocardiocytes, suggesting that soluble molecules were responsible for this transmission. Our study was further supported by the previous study which showed the conditioned medium-mediated transmission of ER stress between tumor cells and macrophages^20^. In this study, we further extended this ER stress transmission phenomenon from tumor diseases to the field of infectious and cardiovascular diseases. Our data were further supported by the observation that CVB3 infection up-regulated the soluble damage associated molecular pattern HMGB1 (High mobility group box 1 protein)^33^, which was also reported as an ER stress inducer^34^. Besides of increased GRP78 in the cardiac macrophages, we also found that GRP78 expression in splenic macrophages was significantly up-regulated in infected mice compared with the control mice (Fig. 2E), indicating that UPR activation could also be induced in macrophages which did not infiltrated cardiac tissue after CVB3 infection. ER stress in splenic macrophages might also be transferred from other splenic tissue cells, just similar with the condition in the infected hearts.

In this study, although the detailed soluble factors involved in this transmissible ER stress are still unclear, previous studies reported that toll-like receptors (TLRs) are involved in the induction of ER stress. They could directly induce ER stress^35^ and synergize with it to promote cytokine production^31^,^36^. More importantly, TLR4 has been reported to potentiate the transmissible ER stress from tumor cells to receiver macrophages^20^. While in our study, the transmissible ER stress seems not dependent on TLR2 or TLR4 pathway. This could be further indirectly supported by the study of Martinon and colleagues^35^, which reported that TLR4 agonists initiated the non-classical ER stress response, mainly triggering the activation of IRE1α pathway but inhibiting the ATF6 and PERK pathways. However, in our study we found that ER stress receiver macrophages underwent obvious ATF and PERK activation. This inconsistence further suggested that TLR2/4 pathway were not associated with this ER stress transmission in the context of CVB3-induced myocarditis. So far, although the exact mechanism of ER stress transmission in CVB3-induced myocarditis is still blur, we found that damage-associated molecular pattern (DAMP)-HMGB1 was robustly increased in the conditioned medium of CVB3-infected myocardiocytes (data not shown). As HMGB1 has been reported to play an important role in initiating ER stress^34^,^37^, it might mediate this ER stress via RAGE-mediated, instead of TLR2/4-meidated pathway. Of course, the exact role of HMGB1 needs to be further elucidated. In addition, other cytokines such as MIF (macrophage migration inhibitory factor) might also participate in this ER stress transmission. It has been reported that MIF could be induced by various virus infection^38^,^39^,^40^ and function as a modulator of toll-like receptors (TLRs) signal pathways^41^,^42^,^43^,^44^, which were associated with ER stress induction^35^. Therefore MIF might also be very likely to associate with this transmissible ER stress between CVB3-infected myocardiocytes and macrophages.

In conclusion, our study showed that in the context of CVB3-induced myocarditis, potent ER stress responses occurred in the cardiac infiltrating macrophages and potentially augmented the inflammation. By deciphering the initiation mechanism of macrophage ER stress, we found that it was not directly caused by the virus stimulation, but was induced after co-culture with the CVB3-infected, ER stressed myocardiocytes. In addition, this ER stress transmission from myocardiocytes to receiver macrophages was mainly mediated by the conditioned medium, but not cell-cell contact way. Our study suggested that the transmissible ER stress from the infected myocardiocytes played an critical role in launching and amplifying pro-inflammatory responses of macrophages, and contributed greatly to pathogenesis of CVB3-induced myocarditis.

## Methods

### Mice, virus and CVB3 infection

Both 6–8 week-old male BALB/c mice and 1–3 day-old neonatal mice were purchased from the Experimental Animal Center of Chinese Academy of Sciences (Shanghai, P. R. China). Mice were housed in a specific pathogen-free room under controlled temperature and humidity at our institution. All animal experiments carried out in this study were conducted according to the Guide for the Care and Use of Medical Laboratory Animals (Ministry of Health, People’s Republic of China, 1998) and the guidelines of the Laboratory Animal Ethical Commission of Soochow University. The protocols were approved by the Ethical Committee of Soochow University. CVB3 (Nancy strain) was maintained by passage through Hela cells (ATCC number: CCL-2). Mice were infected with CVB3 by an intraperitoneal (i.p.) injection with 10^3^ PFU dose of the virus.

### Cell isolation, preparation and culture

BMDMs were prepared as previously described^45^. In briefly, femurs and tibias from BALB/c mice were dissected and bone marrow was flushed out. Cells were filtered through nylon mesh and plated at a density of 2 × 10^6^ cells/ml in RPMI 1640 containing 30% L929 conditioned medium, 10% FBS, 2 mM L-glutamine, 100 U/ml penicillin, 100 μg/ml streptomycin for 6 days. The purity of BMDMs was >90% as determined by FACS analysis using FITC-conjugated anti-F4/80 monoclonal antibody (BD Bioscience) and PE-conjugated anti-CD11b monoclonal antibody (BD Bioscience).

Cardiac infiltrating macrophages were prepared as previously described^11^.

Neonatal mouse myocardiocytes were prepared from 1–3 day-old BALB/c mice as follows: Hearts of 8–10 neonatal mice were extracted, pooled, washed to remove blood and then minced into 0.5–1 mm^3^ pieces in Ca^2+^, and Mg^2+^-free PBS. Myocardiocytes were dispersed by incubating in PBS containing 0.1% of trypsin (Sigma) and 0.05% of collagenase II (Roche) at 37 °C with constant agitation. The supernatant containing released cells was removed every 10 min into an equal volume of cold DMEM complete medium (abandon the first two times) until most of the myocardium was disrupted. Cells were collected and enriched by two sequential pre-plating steps on 90-mm dishes which removed non-myocardiocytes. Cells were incubated at 37 °C with 5% CO_2_ for 48 h and then infected with CVB3 at a dose of 10 MOI.

For co-culture experiments, neonatal myocardiocytes (5 × 10^5^/well) were seed with RAW264.7 (ATCC number: TIB-71) cells or BMDMs (5 × 10^5^/well) in 6-well plates and then infected with CVB3 (10 MOI) for 24 h. And then co-cultured RAW264.7 or BMDMs were isolated by FACS using FITC-conjugated anti-F4/80 monoclonal antibody (BD Bioscience), and ER stress induction was detected.

For transwell assays, neonatal myocardiocytes (2 × 10^5^/well) were added in the upper wells (insert with 0.4 μm pore for 12-well; BD) followed by CVB3 (10 MOI) infection, meanwhile macrophages (2 × 10^5^/well) were added in the bottom wells. Twenty-four hours later, macrophages were collected for the detection of ER stress and pro-inflammatory cytokine.

To prepare conditioned medium, 2 × 10^6^/well freshly isolated neonatal myocardiocytes were plated in 6-well plates and cultured for 48 h, and then the cell monolayers were washed 3 times with PBS and placed in 3 ml DMEM containing 10% FBS followed by CVB3 (10 MOI) infection for 6, 12 or 24 h. The supernatants were collected, centrifuged at 1,000 g for 10 min and stored at −80 °C until using.

### XBP1 splicing analysis

Total RNA was extracted with TRIzol reagent (Invitrogen), reversely transcribed into cDNA with oligo dT primers (Takara, China) following the manufacturer’s protocol. To evaluate relative expression levels of XBP1u/XBP1s, RT-PCR analysis was performed using PCR SuperMix (Invitrogen). XBP1 primer sequences were as follows: 5′-AAACAGAGTAGCAGCGCAGACTGC-3′ and 5′-GGATCTCTAAAACTAGAGGCTTGGTG-3′, which was designed so that the PCR products contained both the spliced form of XBP1 (454-bp) and the unspliced form of Xbp1 (480-bp). GAPDH was used as a loading control, with primers as follows: 5′-GAGCCAAACGGGTCATCATCT-3′ and 5′-GAGGGGCCATCCACAGTCTT-3′. The cDNA fragments were resolved on 2% agarose gels. Gels were stained with 0.5 μg/ml EtBr in 1× TBE for 20 min, washed in water for 20 min and imaged with a gel-imaging system (Tanon). Quantification was based on relative band intensity.

### Real-time PCR analysis

Total RNA was extracted with TRIzol reagent (Invitrogen), reversely transcribed into cDNA with oligo dT primers and then subjected to real-time PCR assays using a Light cycler 480 and SYBR Green system (Roche) following the manufacturer’s protocol. Expression of target genes was normalized to GAPDH, and quantified using the 2^−ΔΔCt^ method.

### Immunofluorescence assays

Mice hearts were immersion-fixed in 10% neutral-buffered formalin overnight followed by embedding in paraffin. Frozen sections about 6 μm thick on glass slides were prepared. For antigen retrieval, the slides were exposed to hot steam for 20 min in 1 mM EDTA, pH 8.0. Mouse heart sections were incubated with a rabbit antiserum to GRP78, at 1:200 (ab108615, Abcam), and rat anti-mouse monoclonal antibody to F4/80, at 1:50 (ab16911, Abcam), at 4 °C for 16 to 18 hours. Appropriate secondary antibodies were applied for 90 minutes at room temperature. Sections were then stained with DAPI to identify nuclei. All the commercial immunochemicals were diluted as recommended by the suppliers. Images captured using the Panoramic Viewer software version 1.14.50 (3DHistech; Budapest, Hungary).

### Western blot analysis

Cells were washed with cold PBS before the addition of an appropriate volume of lysis buffer (50 mM Tris-HCl, 150 mM NaCl, 1% NP40, 0.5% deoxycholate, 0.1% SDS) supplemented with protease inhibitors (Roche). After incubation for 30 min on ice, cell lysates were centrifuged at 12,000 g for 15 min at 4 °C, and protein-containing supernatant was collected, and fractionated by 10% SDS-PAGE followed by transferring onto nitrocellulose membranes. After blocking with 5% non-fat milk-PBST, membranes were respectively incubated with primary antibodies against GRP78, PERK, phopharylated PERK, eIF2α, phopharylated eIF2α, IRE1α (Cell Signaling Technologies), phopharylated IRE1α (Thermo fisher), or ATF6 (Abcam). After washing with PBST, membranes were further incubated with an appropriate HRP-labeled goat anti-rabbit secondary antibody. Detection was carried out by enhanced chemiluminescence (Thermo Scientific Pierce) according to the manufacturer’s instructions.

### Adoptive Transfer of Macrophages

Macrophages of recipient mice were depleted via dichloromethylene diphosphonate (Cl_2_MDP)-loaded liposomes as described previously^11^. The depletion efficiency was >70% as determined by FACS assays. ER stressed BMDMs were prepared by treating with 0.2 μg/ml Tm for 12 h, and then collected and adoptively transferred to the macrophage-depleted recipients at a dose of 5 × 10^6^/mice at day 3 post CVB3 infection.

### Tissue histopathology and myocarditis severity evaluation

Heart tissues were collected at day 7 post CVB3 infection, fixed, embedded in paraffin, sectioned and subjected to H&E staining. Heart sections were examined by two independent investigators in a blinded manner, and the severity of myocarditis was assessed as the percentage of the heart section with inflammation compared to the overall size of the section, with the aid of a microscope eyepiece grid as described previously^46^.

### Echocardiography

Mice were assessed by echocardiography system (Vevo2100, Visual Sonics, Canada) for left ventricular ejection fraction (LVEF) and left heart fractional shortening (LVFS) as previously described^47^.

### Hemodynamic parameters

Systolic blood pressure (SBP), diastolic blood pressure (DBP) and heart rate (HR) were determined in mice using tail cuff method (IITC Life science MRBP Blood pressure system). The blood-pressure analysis system was calibrated prior to each experiment and was programmed to measure average (from 5 consecutive readings) systolic and diastolic blood pressure and heart rate every 5 min.

### Statistical analysis

All data are expressed as mean ± SEM. Statistical significance of differences in 2 groups or more than 2 groups was respectively determined by t-test or one-way ANOVA followed by Bonferroni test using GraphPad Prism version 5.0 (GraphPad Software Incorporated). P values less than 0.05 were considered to be significant.

## Additional Information

**How to cite this article**: Zhang, H. *et al*. Transmissible endoplasmic reticulum stress from myocardiocytes to macrophages is pivotal for the pathogenesis of CVB3-induced viral myocarditis. *Sci. Rep.* **7**, 42162; doi: 10.1038/srep42162 (2017).

**Publisher's note:** Springer Nature remains neutral with regard to jurisdictional claims in published maps and institutional affiliations.
